# The Evolution of Vigilance and Its Atrophy Preceding the COVID-19 Global Pandemic

**DOI:** 10.3389/fpubh.2022.789527

**Published:** 2022-05-18

**Authors:** Theodore J. Witek, Robert Schwartz

**Affiliations:** ^1^Institute of Health Policy, Management, and Evaluation, University of Toronto, Toronto, ON, Canada; ^2^Dalla Lana School of Public Health, University of Toronto, Toronto, ON, Canada

**Keywords:** COVID-19 pandemic, atrophy of vigilance, pandemic preparedness, public health, health policy

## Abstract

**Introduction:**

Recent infectious outbreaks preceding the COVID-19 crisis resulted in the evolution of vigilance for preparedness against the next pandemic. This vigilance was maintained to varying degrees in different jurisdictions.

**Objective:**

To evaluate the evolution of vigilance following previous epidemics and pandemics and the subsequent atrophy of vigilance prior to the COVID-19 global pandemic.

**Methods:**

We evaluated documentation discussing US, Canada, and South Korea from March 2002 to October 2021. Our policy search strategy was rooted in academic literature, government documents and media reports.

**Results:**

In the US, there were examples of atrophy of vigilance; however, there was clear understanding of pandemic readiness actions that were simply not executed amongst political chaos. In Canada, political mishaps were less evident at the time the pandemic unfolded. Nevertheless, atrophy was evident with erosion in preparedness programs following SARS. South Korea appeared least subjected to atrophy of vigilance. The more recent MERS outbreak prompted evolution of sustained vigilance and compliance with basic public health measures such as mask wearing.

**Recommendations:**

Policy options need to be explored and instituted that increase protection of preparedness programs through institutional safeguards and accountability measure.

## Introduction

The past 25 years have seen several disease outbreaks (Table 1) that emerged at the forefront of public concerns. As we experience these outbreaks, several countries along with regional and global public health agencies work to capture learnings on preparedness and execution. Yet failures and missteps nevertheless persist prompting the legitimate question of “when will we ever learn?”

**Table 1:** History of pandemics in the 21st century.

**Virus**	**Date**	**Virus type**	**Natural host**	**Death toll**	**Reference**
COVID-19	2019–Present	Coronavirus	Unknown, possibly bats	6,167,568*	(1)
MERS	2015–Present	Coronavirus	Bats, Camels	858	(2)
Ebola	2014–2016	Ebolavirus	Unknown, possibly bats	11,325	(3)
Swine Flu	2009–2010	H1N1 Virus	Pigs	284,500	(4)
SARS Flu	2002–2003	Coronavirus	Bats, Civets	774	(5)

*As of August 5, 2021.

Public policy scholars offer conceptual frameworks to understand when and why learning and policy change, considering crises, occurs, and is or is not sustained over time. The policy agenda and policy change literature identify disasters as trigger events (6) or focusing events (7) that hold high potential for policy change. In Multiple Streams Framework (MSF) (8) disasters create windows of opportunity for policy change to occur—the framework recognizes that disasters do not necessarily lead to policy change. On the other hand, atrophy of vigilance theory posits that in hazardous systems, disaster will lead to stricter safety measures (9). While both agenda change and atrophy of vigilance hypothesis suggest the possibility of immediate safety restrictions, in response to disasters, they differ in the expected durability. Agenda change approaches contend that change is likely to be lasting involving changes in policy subcommunities and the formation of new institutions (10). Atrophy of vigilance theory suggests that without manifestations of further incidents, vigilance in hazardous systems will begin to relax within 1 or 2 years and within a decade atrophy will be well-advanced (11).

There is some research suggesting that atrophy of vigilance can be prevented even in the absence of further incidents. Building on his analysis of regulatory policy over two decades following the Exxon-Valdez oil spill disaster, Busenberg (12) suggests that sentinel organization, such as government mandated advisory councils, can effectively maintain vigilance over time. The need for ongoing vigilance has long been recognized in “high reliability” domains (e.g., nuclear powerplant, civil aviation) where even low probability errors can cause dire and substantial consequences (13). Professional practice and government regulations have largely succeeded in maintaining vigilance over time and preventing crises (14, 15). What is unclear from the literature is if and when governments can successfully implement institutional safeguards of vigilance, such as sentinel organizations, to maintain high levels of preparedness over time for pandemic crises.

**Figure 1:**
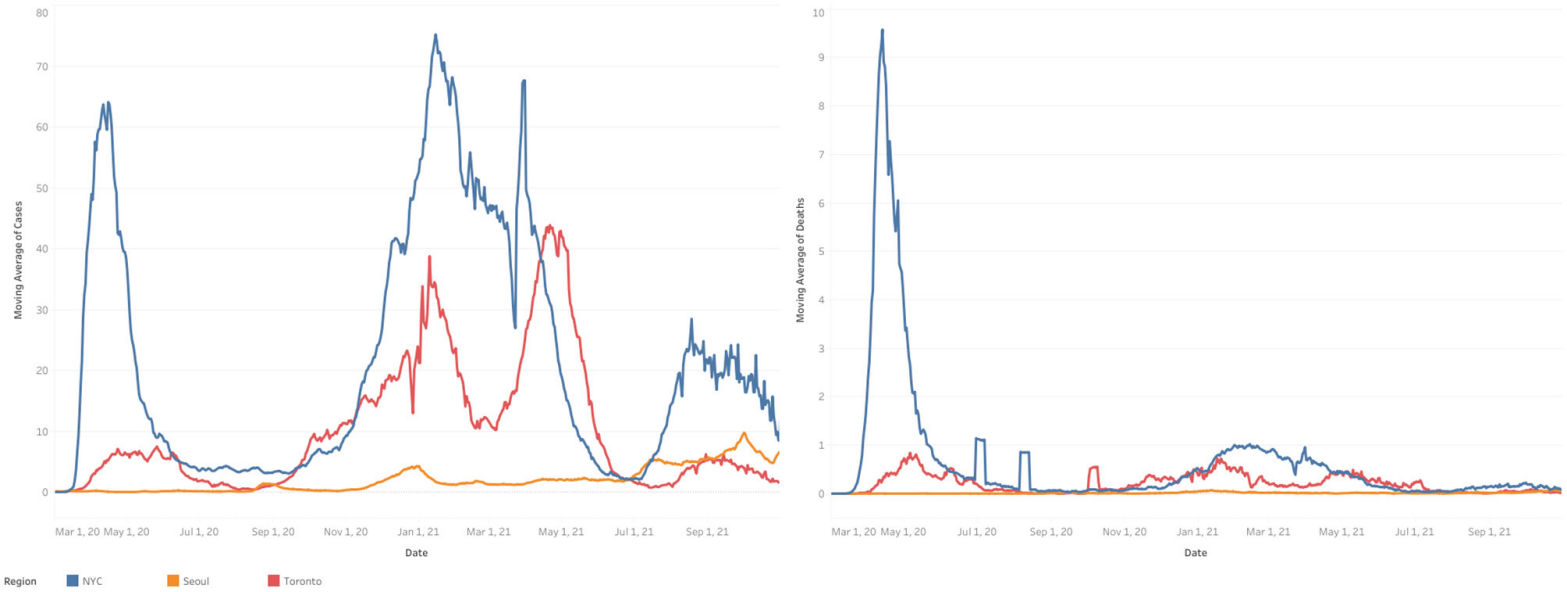
Infection and death rates in New York City, Toronto and Seoul. Rolling 7-day averages of COVID-19 infections and deaths (expressed per 100K) from March 2020 through June 2021 in Seoul, Toronto, and New York City. Source: Data for Toronto Aggregated by COVID-19 Canada Open Data Working Group. Data for Seoul aggregated by Coronaboard (Coronaoard.kr). Data for New York City aggregated by the New York Times. Prepared by Farbod Abolhassani. University of Toronto.

Several sentinel organizations emerged from recent infectious disease outbreaks. Government ability to sustain the focus on pandemic preparedness is a common thread in the devastating outcomes observed (Figure 1).

Recent disease outbreaks that tested public health preparedness include SARS in Toronto in 2002–2003, Ebola, and H1N1 in the United States (US) in 2014 and MERS in South Korea in 2015. Each led to considerable learning and policy change toward developing vigilance reflecting safety values. We explore the emergence of post-breakout vigilance, describe how past experiences brought each jurisdiction to a given level of perceived preparedness and compare the atrophy of vigilance over time. We draw out learnings about how public health organizations and governments can perform better, preventing atrophy in the future.

## Methods

Data were gathered between March 2020 and October 2022, thus beginning with the advent of the pandemic. We monitored major media (with subscriptions for daily alerts from New York Times, Global and Mail, Washington Post) to identify news reports relating to pandemic preparedness in advance of COVID-19. For each of the jurisdictions reviewed, we conducted searches for government reports and academic publications about learning from previous pandemic and measures taken to be prepared for future pandemics. For South Korea, we relied on local key informant who provided reality and accuracy checks to our findings.

## Canada: An Example of Evolution and Atrophy of Vigilance

### Vigilance Evolves

In Toronto, two phases characterized SARS: Phase I affecting mainly healthcare workers, patients, and visitors at four hospitals. Phase II predominately occurred in a single hospital ward. Infections totaled 374 with 44 deaths (16).

Several reports ensued to capture learnings and make recommendations. For example, Svoboda et al. (17) highlighted the importance of surveillance for infections in travelers and adequate surge capacity for emerging infectious diseases. A National Advisory Committee recommended enhancing surveillance mechanisms and public communication strategies, improving coordination within governments and institutions for outbreak containment, and increasing expert involvement (18). Canada's determination to learn from SARS was exemplified by establishing the Public Health Agency of Canada (PHAC), a newly established organization with a clear mandate to anticipate and effectively respond to public health threats.

A detailed analysis of the SARS outbreak was published in the “*Spring of Fear*” (16), emphasizing systemic flaws in the public health infrastructure. The commission also recommended sufficient and sustained public health funding. Furthermore, the report and the National Advisory Committee on SARS emphasized the need for strong emergency PPE stockpiles. These were developed and maintained for the first few years (19) demonstrating the emergence of vigilance.

One of SARS key learnings was the need for early warnings regarding potentially threatening viruses. One of Canada's public health gems—the Global Pandemic Health Information Network (GPHIN) was formed in 1998. This specialized unit scanned the globe for intelligence, detecting and monitoring disease outbreaks as they unfolded. They issued about 1,500 warnings in the past decade (20). In addition to SARS-specific commissions, the *Canadian Pandemic Influenza Preparedness: Planning Guidance for the Health Sector* was published in 2004 and updated in 2018 (21) to include learnings from the H1N1 pandemic. This document discussed goals and objectives for pandemic preparedness, response, and high demand potential for critical resources.

In summary, SARS led to immediate action. Several reports on the SARS response emerged, with the primary document bearing the reality of *fear* in its title. Key areas of focus were identified, and government agencies formed and funded.

### Vigilance Atrophies

With SARS now years in the distance and PHAC functioning for nearly a decade, political changes ensued. The federal government changed a budget bill that functionally transformed the agency's “top doctor” from the agency deputy head to an officer providing scientific advice. This effectively removed responsibilities for staff, budget, and the agenda. Bureaucrats replaced experts.

Extraordinary investigative journalism surfaced atrophy of vigilance fostered by federal health budgetary reform and the suboptimal financing of GPHIN (22). Public health experts serving as a critical warning system for outbreaks had to answer bureaucrats naïve to gathered intelligence (20). Toward the end of 2018, with no imminent pandemic threats, GPHIN was instructed to shift its focus to domestic issues (20), dismantling the pandemic radar.

PPE stockpiles were also not maintained. Supplies diminished in quantity and utility reflecting general atrophy of the vigilance established following SARS. A 2010 audit report noted that officials were uncertain as to stockpile contents with acquisitions driven by available funds vs. comprehensive needs (23). Budgets for stockpiling had also diminished since the post-SARS alertness (24), with reductions in facilities and staff. For example, spending on warehouse leasing was $2.5 million in 2019, down from $7.7 million in 2010–11 (25). A 2019 warehouse closing saw the disposal of 2 million expired N95 masks (24, 26). The fact that physicians were reusing masks during the current pandemic reflects the consequences of atrophy. As described in one editorial on stockpile preparedness: “As the public's memory of SARS faded, the pressure on government to spend money on preparations for the next outbreak faded with it” (27).

The focus away from the intended function of Canada's GPHIN pandemic warning system reflects the erosion of sentinel functions also evident elsewhere. Sentinel organizations such as PHAC were formed after SARS; however, there were pre-COVID-19 warnings on evolution away from its core mandate. Warnings of “mission creep” of PHAC came right before the pandemic. Specifically, a 2019 report highlighted that more than half of PHAC's budget was directed at health promotion and disease prevention potentially overlapping with other organizations. The call for ensuring “PHAC is focused on the new and evolving challenges it will invariably face in its next 12 years of existence” (28) was an eerie predictor of the year ahead.

Erosion from traditional pandemic preparedness was evident elsewhere. For example, a 2019 report on the state of public health in Canada (29) was void of “pandemic,” “outbreak,” or “virus” (30).

## United States: Chaotic Erosion of Established Vigilance

### Vigilance Evolves

Two epidemics occurred between the Spanish Flu crisis of 1918 and COVID-19 pandemic, the latest being the H1N1 influenza outbreak of 2009. This outbreak prompted several reports of lessons learned for preparedness (31, 32), including items such as personal protective equipment (PPE) supply chain (33). A report by the Assistant Secretary for Preparedness Response (ASPR) within the Department of Health and Human Services (HHS) published a 2009 H1N1 Retrospective and Improvement plan (32). For example, under community mitigation measures, it listed states being able to accommodate Strategic National Stockpile (SNS) of PPE as a success and building the evidence base to support guidance on appropriate level of respiratory protection as an opportunity for improvement.

**Figure 2:**
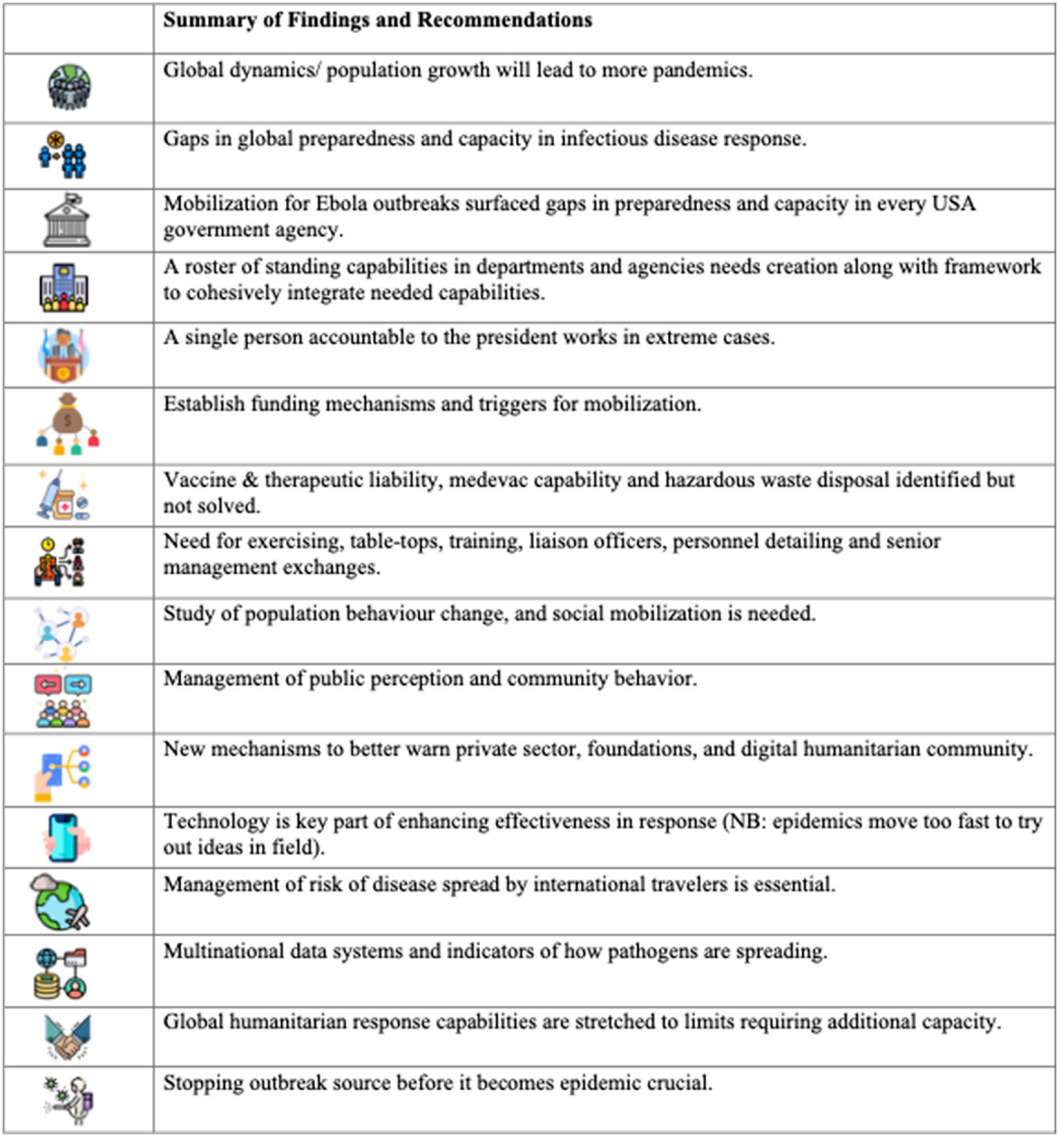
Recommendations from National Security Council. Icons made by “surang”, “Pixel meetup”, “Eucalyp”, “Freepik”, “Skyclick”, “prettycons”, “geotatah”, “photo3idea_studio”, “juicy_fish”, “max.icons”, “Flat Icons”, “ultimatearm”, “small.smiles”, “Becris” from www.flaticon.com.

In addition to the H1N1 outbreak, the US had experienced a terrifying Ebola threat in 2014. That also contributed to the period's evolution of vigilance. Despite only two transmission cases inside the US, a poll showed Americans ranking Ebola as the third-most-urgent health problem facing the country (34). After Ebola, the White House quickly established a National Security Council (NSC) office to lead in both the acute situation of a pandemic and building preparedness capacity (35). Concerns were exemplified in an NSC memorandum listing several alerts, including gaps in preparedness and capacity surfacing in every major government agency tasked with health and security (36). These are summarized in Figure 2.

Additionally, the DHS conducted annual updates since 2005 on pandemic planning, analyzing transportation systems and health care facilities. In the 2015 report (37), authorities were warned that the healthcare and public health sector would have to prioritize limited resources and warned of potential significant shortages in vaccines, antivirals, pharmaceuticals, PPE, and equipment including ventilators. The report noted that the 2009 H1N1 pandemic saw demand outpace supplies.

As Ebola was emerging, the Obama administration refocused and reinforced NSC global surveillance which was institutionalized as the Directorate for Global Health and Security and Biodefense (38). The aim was fast and efficient government response to heath security threats. Late in 2016, an executive order also advanced the Global Health Security Agenda (39). The NSC directorate was dissolved in <2 years by the new Trump administration; however, new initiatives followed. For example, in May 2019, a Global Health Security Strategy was released, defining the actions necessary to “prevent, detect and respond to infectious disease threats” and reconfirming support of the GHSA (40). Support for the GHSA was extended to 2024.

A 2019 report (41) discussing these initiatives, expressed concern about a leadership gap in the White House, noting the lack of clarity regarding whom would be in charge if a grave pandemic threat emerged. The importance of navigating challenging political issues like quarantine and travel bans and in communicating and reassuring the public was deemed insufficient.

From January 2019, a year before the pandemic, through August, the Federal Administration conducted scenario planning, termed “*Crimson Contagion*.” A draft report dated October 2019 (42) laid out “just how underfunded, underprepared, and uncoordinated the federal government would be” for the situation that was about to face them in a matter of months (43). The scenario exercise foreshadowed the ensuing chaos of the US response to COVID-19 in an almost chilling manner: “Federal agencies jockeyed over who was in charge, State officials and hospitals struggled to figure out what kind of equipment was stockpiled or available. Cities and states went their own ways on school closings” (43).

One final act of preparedness that gained some media attention on its lack of utility during the COVID-19 crisis was a 2016 pandemic playbook with stepwise prioritization of key functional areas (44). The playbook noted areas of communication, diagnostic capacity, case detection, and guidance on federally led procurement of key material such as PPE. In 2017, Trump administration officials were briefed on the playbook.

### Vigilance Atrophies

Pandemic preparedness was a priority after Ebola. Yet, for COVID-19, there was a gaping void from lack of follow through (35). While we describe clear examples of the atrophy of vigilance, the US also appeared to be characterized more by political chaos driving a lack of execution. While pandemic planning always seemed to be playing out on center stage, the theater appeared to be largely empty. Gaps in preparedness were identified in report after report. The recommendations and warnings in the DHS memo (36) were spot on. Sadly, the same list was valid as the COVID-19 pandemic approached a year later.

In January 2017, the Trump administration was provided briefing papers and participated in a transition exercise on pandemic threats. The outgoing administration deliberately chose a pandemic exercise given its threat to public health and national security (45). Unfortunately, much of the assistance offered was discarded by the incoming team (46).

The federal government distributed more than 85 million N95 respirators to assist with the 2009 H1N1 influenza pandemic. However, the stockpile supply of N95 respirators was not replenished. As pointed out by Busenberg (47), this was a pronounced atrophy of pandemic vigilance reflecting diminished political attention.

A central hub of global vigilance was the GHSA. Congress allocated $1 billion for the GHSA until 2019 when funding for global health security was halved. The Trump administration's FY2020 request for global health security was even less.

While there were increases in CDC staff abroad following the Ebola crisis, 2018 reductions followed decreased funding for the GHSA. Another short-sighted symptom of atrophy came in the summer of 2019 when a resident advisor to the US Field Epidemiology Program in China, was returned to the US. Taylor (48) reported that the CDC's China headcount had shrunk to around 14 staffers, from ~47 people since Trump took office.

DHS yearly updates, which modeled the havoc that a pandemic would wreak on the country's infrastructure were stopped in 2017 due to bureaucratic debate on their value and even worse, could not be readily located when COVID-19 hit the country (49). Analysts and supercomputers at several national laboratories were no longer in action, with significant capabilities allowed to atrophy and decay (49). The Crimson Contagion simulation (42) demonstrated considerable knowledge about pandemic risks and the types of challenges were accurately predicted. The failure to address the shortcomings is troublesome, particularly since sufficient preventive policy and funding attention was lacking, while disorganization ensued in the reality of the COVID-19 pandemic.

The pandemic playbook was evidently not part of the plans of authorities when faced with the emerging crisis (50). The playbook cited the need for “unified messaging” on federal response and early coordination of risk communication through a single spokesperson. This was mishandled entirely with non-expert, contradictory, and, at times, mind-boggling communication (51). Additionally, the playbook stressed the need for confidence on case detection and maintaining diagnostic capacity. What the country faced was an initial faulty CDC test which slowed mass screening capacity (52). If the playbook were followed there would have been federally led procurement and deployment of PPE in January. However, 5 months into the pandemic, a dearth of supplies hampered healthcare professionals. A federally led unified response plan detailed in the pandemic playbook was unheeded, and failing the nation.

## Observations From Korea

South Korea faced a respiratory infection outbreak in 2015 with the MERS, involved 186 cases with 38 fatalities. Headline statistics related to the 83% of transmission events due to “superspreaders” and that 44% of the total cases represented patients whose exposure was a nosocomial transmission at 17 hospitals (53). Economic impact was felt heavily with 2.6 billion USD lost in tourism revenue alone. This experience impacted their preparedness when COVID-19 arrived in early 2020.

### Vigilance Evolved

MERS-CoV was still a painful memory in 2020 which likely contributed to an early and aggressive response to COVID-19, including the relative high willingness to adhere to public health advice. Face mask wearing was also fostered by local and cultural considerations ranging from their consistent and frequent use against air pollution, particularly the seasonal “yellow dust” (54) as well as fashion-driven compliance (55).

The Division of Public Health Crisis Response in Korean Centers for Disease Control and Prevention (KCDC) was established in 2007 to lead against a national public health crisis due to emerging infectious disease. This followed WHO's post-SARS call for country-level pandemic preparedness and planning (56). Their national disaster plan outlined general actions for a variety of phased situations. The plan also detailed the composition and role of a Central Human Infection Countermeasure Squad CHICS)—a group that serves as a control tower to manage a crisis—even pre-emptively should an emerging infectious disease surface abroad.

The 2015 MERS outbreak prompted a comprehensive report rooted in broad-based stakeholder feedback and an outline of the prevention system's reform. Detailed were 48 tasks to prevent influx of infectious disease, early detection, case confinement, and improvements to the medical environment and response system (57). This included designation of 20 tertiary regional infectious disease hospitals, specially equipped with negative pressure isolation capabilities. While the ratio of hospital beds per capita in South Korea is high, reducing the pressure in “flattening the curve” (58), there was a benefit to addressing infrastructure details. Furthermore, cultural-driven behaviors of nursing care and visitation of sick, particularly in the years preceding the COVID-19 pandemic, were given attention. The report also spoke of the importance of management skills.

Several methods of response that are unique to South Korea and believed to limit transmission and impact are detailed by Kang et al. (59). Unlike the US CDC testing failure (60), South Korea capitalized on an ongoing project started in 2017 at their CDC which was aimed at general coronavirus detection, then narrowed to focus on the new SARS-CoV-2.

Through the government's Infectious Disease Control and Prevention Act, tracking and isolation took on elevated vigilance. Case tracking was allowed *via* seven categories of data including credit cards, cell phone GPS, and security camera recordings (59) with emergency alerts deployed for new cases to alert potential contacts to undergo infection testing. While Korea's Personal Information Protection Act of 2011 in principle bans such collection, use and disclosure, the MERS outbreak triggered amendments to the Contagious Disease Prevention and Control Act which override certain PIPA provisions regarding infected individuals and those suspected of being infected (61). Many believe that a social consensus was achieved among most people on transparent information sharing for public cooperation after MERS (59, 62).

## Vigilance Sustained

The temporal proximity to MERS CoV was evident in the public's behavior. Indeed, a 2020 poll revealed higher level of individual adherence to public health measures with COVID-19 than observed for MERS CoV in 2015 (63). This included discouragement of all social gatherings and social distancing which had good voluntary compliance when the pandemic manifested (62). The facemask market in South Korea grew steadily from 2015 from 0.67 trillion won to an estimated 1.8 trillion won in 2020 (~1.6 billion USD) (64).

***S***outh Korea entered 2020 with among the most hospital beds per person among OECD countries, with intensive care beds near its average (58). The country also benefited from post-MERS infrastructure investments including increased negative pressure isolation wards. While these facilities were rapidly utilized during caseload peaks, health system leaders were credited with mobilizing regional reorganizations of health systems parallel to hospital-level interventions that concentrated and allocated resources (65).

Public-private partnerships emerged as exemplary as community treatment centers opened in the training facilities of private companies such as Samsung and LG. Fifteen such community centers admitted over 3,000 patients within 3 weeks of March 2020 (65).

Innovative operation strategies are central in abating a crisis such as COVID-19 and this feature was evident concerning testing. Having an RT-PCR test with results in 6 h is evidence of such. This was available to 18 locations on January 31, 2020. Within a month, drive-thru testing centers were opened which afforded less opportunity for cross-infection, less waiting, and testing time, and fewer changes of PPE per patient. This system was initially discussed in 2018 in the context of potential bioterrorism responses to deliver antidotes (59). Such innovative drive and walk-through centers, rapid processing of tests even down to 4 min, was praised internationally (66).

Advanced testing kits were a central component of mitigation strategies. It is apparent from reports that MERS served as a tangible catalyst in building future virus testing technology. There was not the CDC reliance as in US but rather an aggressive enlistment of the private sector. South Korean officials urged their involvement at the end of January with test ramping up (10,000/day in a population of 50 million) within a month (67). In summary, testing was identified as a key area of focus with no atrophy of preparedness evident.

Rapid application of technology was evident at the very beginning of the pandemic. On February 10, 2020, the Central Disaster Management Headquarters of Korea announced a self-diagnosis app to monitor all incoming travelers through a special entry procedure (66). This required consent to use the app which connected both travelers and citizens to subsequent offerings of health information. For most, this personal privacy opening was accepted as a reasonable price for health and well-being. In fact, a survey in early March 2020, 78% of respondents agreed that human rights protections should be eased to strengthen virus containment efforts (67). The practices were nevertheless the subject of a healthy debate (68, 69). By March of 2020, the scope and detail of disclosed information on cases and contacts curtailed by the KCDC (70).

In summary, South Korea was relatively well-organized with a palpable degree of readiness (71), perhaps due, in part, to the experience of MERS that was closer in proximity to COVID-19 than SARS in Toronto and more widely felt than the limited health and social impact of Ebola in USA. Finally, the societal willingness in compliance with preventive measures and acceptance of tracking and tracing technology proved impactful.

## Discussion

The principal findings of our report are that the US showed clear examples of atrophy of vigilance; however, there was clear understanding of pandemic readiness actions that were simply not executed amongst political chaos (51). In Canada, political mishaps were less evident at the time the pandemic unfolded (72). Nevertheless, atrophy was evident with erosion in preparedness programs following SARS. South Korea appeared least subjected to atrophy of vigilance. The more recent MERS outbreak prompted evolution of sustained vigilance and compliance with basic public health measures such as mask wearing (59).

The strength of our approach is the direct comparison across three jurisdictions which had similar evolution of vigilance in pandemic readiness but varying degrees of atrophy prior to COVID-19's manifestation. This allowed for deeper insights into potential reasons for atrophy and thus, recommendations for the future. Our selection of countries was based on those experiencing measures against a relatively recent pandemic that prompted an evolution of preparedness before the manifestation of COVID-19. Additionally, they were deemed most appropriate as each had sentinel institutions responsible for preparedness and all three of the selected countries had sufficient documentation of efforts. While USA and Canada were the subject of more investigative journalism than South Korea, the later added a unique perspective with respect to the relation of the population to government as discussed below. Finally, several of the reports in our analysis were based on a single jurisdiction, particularly in local media and governmental bodies, thus, limiting broader comparisons, insights, and recommendations.

In alignment with what both *Multiple Streams Framework* and *Atrophy of Vigilance hypothesis* would predict, Canada, the US and South Korea all rallied to safety values following 21st century global and domestic major infectious disease outbreaks. Expert commissions created comprehensive reports with detailed recommendations for preventing and mitigating harm from future infectious disease events. New institutions were established in all three countries. Elaborate pandemic preparedness plans, which included governance and leadership structure, were created with stipulations for continuous updating. All countries developed emergency PPE stockpiles. Additionally, global intelligence-gathering and surveillance systems were set-up and functioning. In summary, vigilance followed from previous crises.

Atrophy of vigilance is partially responsible for inadequate COVID-19 response in Canada and the US; South Korea mostly retained its vigilance. A combination of explanatory factors emerges from our exploratory research: (1) natural atrophy over time in accordance with Atrophy of Vigilance Theory; (2) political and socio-political influences; (3) sentinel organization performance.

### Natural Atrophy

Busenberg (9) posited that vigilance following a crisis atrophies over the course of 10 years. SARS hit Canada in late 2002–18 years before COVID-19. H1N1 arrived in 2009, however, it caused relatively small damage as measured by Canadian morbidity, mortality, and economic loss. Ebola emergence in the US in 2014 and MERS in Korea in 2015 were not felt domestically in Canada. In the years following SARS, the need for pandemic planning was felt strongly in governmental and non-governmental organizations. By 2020; however, it was not on the radar outside of a small group of specialized public health professionals and academics. Atrophy in Canada followed the theory's predicted course. In the US, domestic experience with H1N1 occurred 10 years prior to COVID-19 but was followed by a serious infectious disease threat—Ebola—occurring just 6 years before COVID-19. Atrophy of Vigilance theory would predict that insufficient time had elapsed for atrophy to have set-in. Indeed, pandemic preparedness was so high on the radar of the outgoing Obama Administration in 2016 as to make the focus of a transition meeting with the incoming administration. Furthermore, a major pandemic response exercise was executed in the year prior to COVID-19. The natural course of atrophy then should not have had as large an effect as experienced. Thus, one can appreciate that the effect of government denial and minimization loomed large in the United States (73). South Korea's major losses suffered from MERS in 2015 alongside ongoing infectious disease threats in the region meant that atrophy did not have a chance to set in before the arrival of COVID-19.

### Political and Socio-Political Influences

Jurisdictions with less communitarian cultures and more inclined to individualism would be expected to devote less resources to safety concerns that are not perceived to pose immediate threats. Individualism mixed with libertarian tendencies, as in large swaths of the US and as fired up by Trumpism appear to have created the perfect storm for disregarding careful public health pandemic planning, limiting government spending on preparedness, and for not paying heed to early warnings of the emerging pandemic. South Korea is close to the opposite end of the continuum. It is highly communitarian and South Koreans both rely on the government for their safety, social and physical security (74).

Moreover, South Korean's culture reflects a deep trust of authorities and a deep sense of honor connected perhaps with the Confucian culture and mandatory military service. Canada is somewhere between the US and South Korea on this continuum. It more resembles European social democracies than the US while having segments of the population with libertarian views. Notably, during a key period after SARS and before COVID-19 (2006–2015), Canada was led by Stephen Harper's government which intended to decrease government intervention. During this period, much, but not all, of Canada's atrophy of vigilance occurred. Justin Trudeau's Liberal government, elected in 2015, did not undo much of the Harper era's damage to pandemic preparedness.

### Sentinel Organization Performance

Seminal studies of atrophy of vigilance notes the key role of sentinel organizations. A sentinel organization is a permanent and independent institution devoted to maintaining vigilance through ongoing monitoring and ensuring continual learning about changing contexts to that vigilance and be adjusted as needed (75).

PHAC, established in the wake of SARS, appears to have the trappings of a sentinel organization. Indeed, it functioned as such for many years by collecting intelligence, issuing warnings, and leading pandemic preparedness planning. What PHAC lacked was sufficient independence. Political forces under the Harper government were able to replace public health professionals with appointees who had little understanding of pandemic preparedness or appreciation of PHAC's role as a sentinel organization. The investigative reporting on the GPHIN made this atrophy visible (20, 22, 76). Furthermore, warnings of agency “mission creep” and a call for PHAC mandate and spending review were clearly on target; however sadly too late to execute before COVID-19 arrived (29, 30).

Similarly, the US established what appeared to be a sentinel organization in an office in the National Security Council (NSC) whose role was to ensure pandemic preparedness and to lead execution in the case of a pandemic (77). The DHS assumed the role of an apparent second sentinel organization charged with ongoing monitoring and annual reporting on pandemic planning. While both organizations performed their monitoring and reporting duties well, they were disarmed in affecting execution.

Also in the US, the Office of the Assistant Secretary of Preparedness and Response (ASPR) operates within the HHS and has the mission to lead the nation's medical and public health preparedness for response to, and recovery from disasters and public health emergencies (78). They lead the ongoing coordination of the COVID-19 response across HHS.

Investigative reports noted that the agency was drastically underfunding medical responses to counter pandemics. Furthermore, HHS noted Congress continued to underfund strategic national stockpile minimum requirements. With many potential disaster scenarios under its responsibility looming government was spending more for cybersecurity and missile threats (79). Coming back to the role of sentinel organization, in the context of vigilance, the pandemic concerns appeared to be overtaken by other potential disaster emergencies.

As in Canada, political decision-makers chose to dilute pandemic preparedness resources and to ignore warnings of PPE stockpile depletion and of early signs of the emergence of COVID-19. Arguably, these organizations in Canada and the US lacked the degree of independence necessary to perform as true sentinel organizations. In the case of Canada's GPHIN system, their scientists have subsequently proposed to be based in a university system to gain such independence (80). In the US, a special cabinet position for public health has been suggested to partition efforts away from the HHS big attention and budget getters like Medicare and Medicaid (81).

In liberal democracies, like the US and Canada, the power of the purse and the power to act rests with political masters rather than with technocratic officials no matter how much scientific power is embedded in the bureaucracy. Can such societies find a better path by developing strong sentinel organizations that can maintain long-term vigilance and to resist the whims of politicians? One path may be to require special majorities to override their advice. Another would be to make them accountable directly to the legislature rather than to the executive.

While retrospective assessments of the COVID-19 pandemic will undoubtedly highlight key areas of focus that are fundamental to pandemic management, deliberate attention should be dedicated to uncovering new ways in which vigilance can be institutionalized to temper the ability of politics to disrupt it. We know what to do; however, it should be possible to safeguard institutions that aim for vigilance to protect the population from major threats.

## Conclusions

Vigilance in future pandemic preparation evolved in the countries assessed, with its atrophy evident to various degrees before the COVID-19 global pandemic. To avoid atrophy of vigilance for future pandemics, policy options need to be established that increase protection of preparedness programs through institutional safeguards and accountability measures for key defined areas that are made available both to governmental agencies and the public. It remains to be seen how the vigilance following COVID-19 is re-established for future outbreaks; nevertheless, future reports must focus on sustainability of vigilance and insuring independence of sentinel functions.

## Data Availability Statement

The original contributions presented in the study are included in the article, further inquiries can be directed to the corresponding author/s.

## Author Contributions

Both authors listed have made a substantial, direct, and intellectual contribution to the work and approved it for publication.

## Conflict of Interest

The authors declare that the research was conducted in the absence of any commercial or financial relationships that could be construed as a potential conflict of interest.

## Publisher's Note

All claims expressed in this article are solely those of the authors and do not necessarily represent those of their affiliated organizations, or those of the publisher, the editors and the reviewers. Any product that may be evaluated in this article, or claim that may be made by its manufacturer, is not guaranteed or endorsed by the publisher.
